# Quality in intensive care units: proposal of an assessment instrument

**DOI:** 10.1186/s13104-017-2563-3

**Published:** 2017-06-26

**Authors:** Alexandre Guilherme Ribeiro de Carvalho, Ana Paula Pierre de Moraes, Lilian Maria Sobreira Tanaka, Renato Vieira Gomes, Antônio Augusto Moura da Silva

**Affiliations:** 1 Department of Public Health, Federal University of Maranhão, Rua Barão de Itapary, 155, Centro, São Luís, MA CEP: 65020-070 Brazil; 2 Intensive Care Unit, UDI Hospital, Av. Professor Carlos Cunha, 2000, Jaracaty, São Luís, MA CEP: 65076-820 Brazil; 3 Intensive Care Service, Tarquínio Lopes Filho Hospital, Praça Neto Guterres, 2, Madre Deus, São Luís, MA CEP: 65026-040 Brazil; 4 Nutritional Therapy Multidisciplinary Staff, Copa D’Or Hospital, Rua Figueiredo de Magalhães, 875, Copacabana, Rio de Janeiro, RJ CEP: 22031-011 Brazil; 5 Department of Research, Unimed Rio Hospital, Av. Ayrton Senna, 2550, Barra da Tijuca, Rio de Janeiro, RJ CEP: 22775-003 Brazil

**Keywords:** Structure, Process, Outcome, Quality, Assessment, Intensive care unit

## Abstract

**Background:**

There is an increasing need for standardized instruments for quality assessment that are able to reflect the actual conditions of the intensive care practices, especially in low and middle-income countries. The aim of this article is to describe the preparation of an instrument for quality assessment of adult intensive care services adapted to the actual conditions of intensive care in a middle-income country and comprising indicators validated in the literature.

**Methods:**

The study consisted of five steps: (1) a literature survey; (2) a discussion with specialists by consensus method; (3) a pilot field test; (4) a description of indicators; and (5) an elaboration of the final version of the instrument. Each generated indicator was attributed a score (“out of standard” = 0; “below standard” = 1; “standard” = 2) that allowed calculation of the total score for each service assessed.

**Results:**

A total of 62 indicators were constructed, distributed as follows: 38 structure indicators (physical structure: 4; human resources: 14; continued education and training: 2; protocols and routines: 12; material resources: 6); 17 process indicators (safety: 7; work: 10); and seven outcome indicators. The maximum possible total score was 124.

**Conclusions:**

Possible future applications of the instrument for the assessment of intensive care units that was constructed in the present study include benchmarking, multicenter studies, self-assessment of intensive care units, and evaluation of changes resulting from interventions.

**Electronic supplementary material:**

The online version of this article (doi:10.1186/s13104-017-2563-3) contains supplementary material, which is available to authorized users.

## Background

Within the field of the health sciences, “to assess” means to perform a value judgment of a health program, service, intervention or any of their components in a manner that contributes to decision-making [1]. Intensive care units (ICU) are services that unite several fields of knowledge, technologies, and diagnostic and therapeutic methods; within such a context, evaluations are highly relevant [2].

One of the main uses of assessments is to promote the improvement of the care provided at a given unit. Scientific evidence derived from previous experiences with the implementation of systematic collection of quality indicators shows that it is associated with improving the performance of the corresponding services [3]. Therefore, critical evaluation of processes and outcomes is a crucial step in the improvement of health services.

Despite being widely used in the United States (US), health evaluations were not immediately accepted in Latin America, where their adoption only gained momentum starting in the 1990s. Several factors were adduced to account for that fact, including local economic and social conditions, a lack of specialized professionals, and a strong culture of authoritarianism and clientelism that profoundly continues to this day [4].

Different from the tradition of evaluation in the US, which has been historically centered on the technical quality of healthcare in the hospital setting [5], in Latin America as a whole and, more particularly, in Brazil, evaluation most often focuses on primary care and outpatient programs and services—principally, the ones corresponding to the public sector. Consequently, several relevant programs and countless features related to in-hospital care are not included in such evaluations, despite receiving the lion’s share of the healthcare budget [6].

Several countries, such as the Netherlands, France, Spain, Italy, and Germany, as well as the joint work of study groups from the European Society of Intensive Care Medicine (ESICM) and the Society of Critical Care Medicine (SCCM), have formulated quality indicators and systematized instruments to assess performance in the intensive care medicine setting [2, 3, 7–9]. Although many such indicators have global application, there is an increasing need for standardized local instruments for quality assessment, including indicators robustly adapted to legislation that are able to reflect the actual conditions of the practices applied in low and middle-income countries. Nevertheless, more than 50 years have passed since assessment tools have started to be applied into practice; however, the public policies supporting the formulation and routine use of standardized instruments for the assessment of healthcare services are still scarce, as are the currently available modalities for using the information gathered to date [10].

The aim of the present article is to describe the preparation of an instrument for the assessment of adult intensive care services adapted to the predominant norms in Brazil, while also including indicators validated in the international literature on this subject.

The present study is justified by the need for assessment tools capable of providing information on the actual state of healthcare practices, their relationship with current local norms and the results achieved by intensive care medicine. These authors expect that the information thus produced will be crucial for meeting new challenges, as well as for the formulation of novel strategies for healthcare improvement, by lending support to the decisions made by managers and thus improving the health care provided to the users of these services.

## Methods

The preparation of the assessment instrument lasted from January to November 2013. The process of elaboration consisted of five steps: (1) a literature survey; (2) a discussion with specialists; (3) a pilot field test; (4) a description of indicators; and (5) the formulation of the final version of the instrument.

Some of the indicators included in the assessment instrument were identified through a survey of the specific literature on the subject in the *PubMed* and *SciELO* databases using the keywords “*intensive care unit,” “structure,” “process,” “outcome,” and “quality*”; the search was limited to articles published after 1995. Other indicators were formulated based on norms currently established in Brazil, such as Normative Instruction no. 4 and the Collegiate Board of Directors Resolution (Resolução da Diretoria Colegiada—RDC) nos. 07, 26, and 50. Criteria susceptible to measurement and representative of their corresponding attributes were formulated relative to those norms.

Whenever it was possible, the “standard” considered for each investigated attribute was its presence within or above the minimum cutoff point indicated by the norm. Whenever an attribute was not measured or was absent, it was classified as “out of standard.” The assessment instrument also included a third criterion (“below standard”) for the attributes that were present or measured but were below the minimum cutoff point established by the norm. The criteria were attributed scores (“out of standard” = 0; “below standard” = 1; “standard” = 2) so that, at the end of data collection, the total score of each investigated service was calculated, as was its performance in the various investigated dimensions. The use of terms “norm,” “criterion,” and “standard” in the present study follow Donabedian’s description [11].

Following their formulation, the indicators were divided into categories: structure, process and outcome [4]. Each category was divided into subcategories as needed: structure into the subcategories physical structure, human resources, continued education and training, protocols and routines, and material resources and process into the subcategories safety processes and work processes.

After this first stage, on September 17, 2013, in São Luis, Maranhão, Brazil, a panel of doctors bearing official accreditation in intensive care medicine and working at public or private ICUs in Maranhão met to discuss the initial version of the instrument designed for data collection. The aim of that meeting was to introduce eventual modifications and additional questions or to remove indicators as needed. Only the criteria thus reviewed that achieved 100% consensus among the specialists were retained in the document. At the end of the meeting, all the participants signed the minutes in which the results of the discussion were recorded.

Next, a pilot study was conducted in which the consensus form was applied at three ICUs located in other Brazilian states (two in the city of Rio de Janeiro, Rio de Janeiro, and the third in Florianópolis, Santa Catarina), selected based on convenience sampling (invitation and voluntary acceptation to participate). The ICU were invited taking into account the different stages of development they were in, in order to test the discrimination capability of the instrument (calibration). The pilot study also aimed to assess the need for adjustments, additional changes, inclusions, and exclusions changes, to test whether application of the instrument was feasible and time spent on filling it. For that purpose, a fill-in manual, aiming to guide researchers during interviews, was prepared.

## Results

Table 1 shows publications taken into account for the construction of the current assessment tool [2, 3, 7–9, 12–20].

**Table 1:** Authors, year of publication and indicators used in the construction of the assessment tool for adult intensive care units

Authors	Year	Selected indicators
Ashton et al.	1997	Readmission to the ICU
Berenholtz et al.	2002	Protocols for effective management of pain, sedation, appropriate use of blood transfusions, prevention of VAP, gastrointestinal bleeding and venous thromboembolism; average ICU LOS; readmission to the ICU; catheter-related bloodstream infection rate; patient/family satisfaction assessment
Pronovost et al.	2002	Availability of intensivist staffing
Pronovost et al.	2003	Protocols for sedation, appropriate use of blood transfusions, VAP, gastrointestinal bleeding and VTE prophylaxis
Souza et al.	2006	Patient/family satisfaction assessment; permanence of visitors and family in the ICU
de Vos et al.	2007	Availability of intensivist staffing; bed-to-nurse ratio; patient/family satisfaction; standardized mortality; unplanned extubation rate; ICU LOS
Najjar-Pellet et al.	2008	Protocols for catheter-related bloodstream infection prevention; monitoring of adverse and sentinel events
UçKay et al.	2008	Protocol for VAP prevention; HAI vigilance
Neves et al.	2009	Patient/family satisfaction; permanence of visitors and family in the ICU
Braun et al.	2010	Protocols for management of sedation and analgesia; strategy for lung protective ventilation; adequate antibiotic use; scheduled meetings with relatives; availability of ICU specialists
Kim et al.	2010	Multidisciplinary rounds
Lobo et al.	2010	Catheter-related bloodstream infection rate
Martin et al.	2011	Scheduled meetings with relatives; catheter-related bloodstream infection rate
Rhodes et al.	2012	Availability of a consultant-level intensivist; monitoring of adverse and sentinel events; multidisciplinary rounds; standardized mortality ratio; ICU readmission rate; unplanned extubation rate; catheter-related bloodstream infection rate
McHugh et al.	2013	Bed-to-nurse ratio

*ICU* intensive care unit, *VAP* ventilator-associated pneumonia, *LOS* length of stay, *VTE* venous thromboembolism, *HAI* hospital acquired infections

**Fig. 1:**
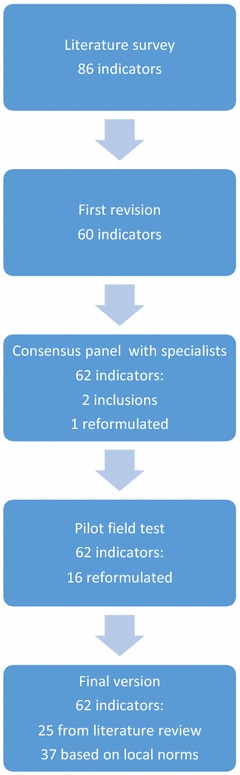
Number of indicators at each stage of the construction process of the assessment tool

Figure 1 shows the number of indicators at each stage of the construction process of the assessment tool.

The first version of the instrument was expanded based on the indicators mentioned in the literature and those included in the norms currently in use in Brazil. That instrument included 86 indicators distributed across the various dimensions. After this first stage, the authors made a revision of all 86 previously listed indicators. That revision resulted in 26 exclusions, leaving 60 indicators to move to the next stage.

The consensus meeting included nine specialists in intensive care. At that meeting, all 60 indicators listed in the previous stage reached 100% consensus and two further indicators were added to the instrument. A total of 62 indicators was reached, 25 of them derived from literature review and 37 new ones built based on local norms. The description of one indicator was reformulated to improve its understanding and to reduce the odds of subjective interpretation by the respondents. On the dimension outcome, answer option “()—Not reported” was added for the cases in which an attribute was measured and available for consultation but was not reported to the interviewer due to administrative reasons intrinsic to each particular institution. It was further decided that responses indicating that option would not be included in the calculation of the final score of the corresponding service, and said indicator was excluded from the overall count.

Table 2 describes the distribution of each section of the final version of the instrument across the various dimensions and their maximum scores; the maximum possible score was 124.

**Table 2:** Distribution of scores among the various dimensions, the numbers of indicators, and the maximum scores on the quality assessment instrument

Dimension	No. indicators	Maximum score
Structure	38	76
Physical structure	4	8
Human resources	14	28
Continued education and training	2	4
Protocols and routines	12	24
Material resources	6	12
Process	17	34
Safety processes	7	14
Work processes	10	20
Outcome	7	14
Final scores	62	124

**Fig. 2:**
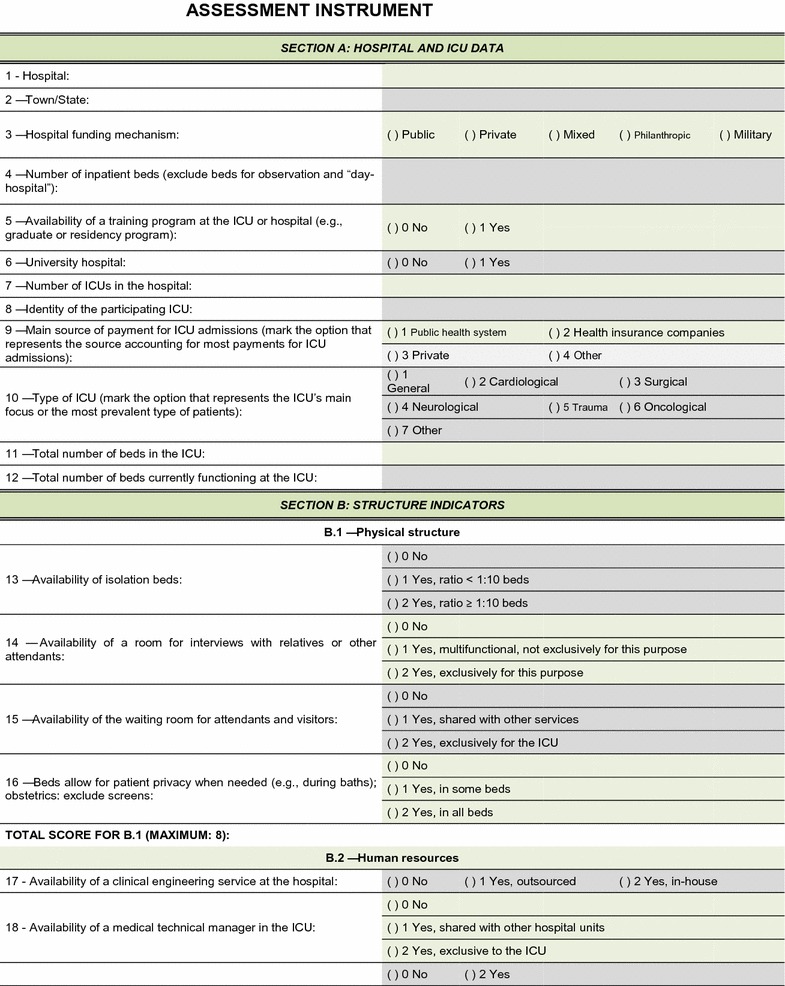
Final version of the quality assessment instrument

**Fig. 3:**
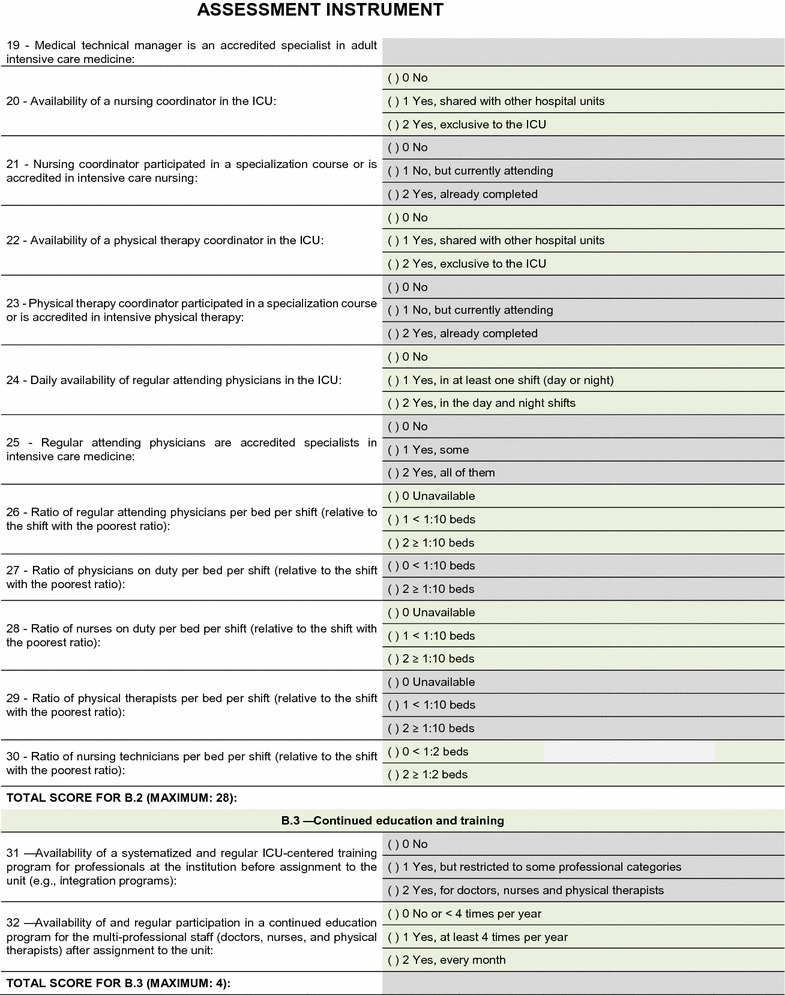
Final version of the quality assessment instrument

**Fig. 4:**
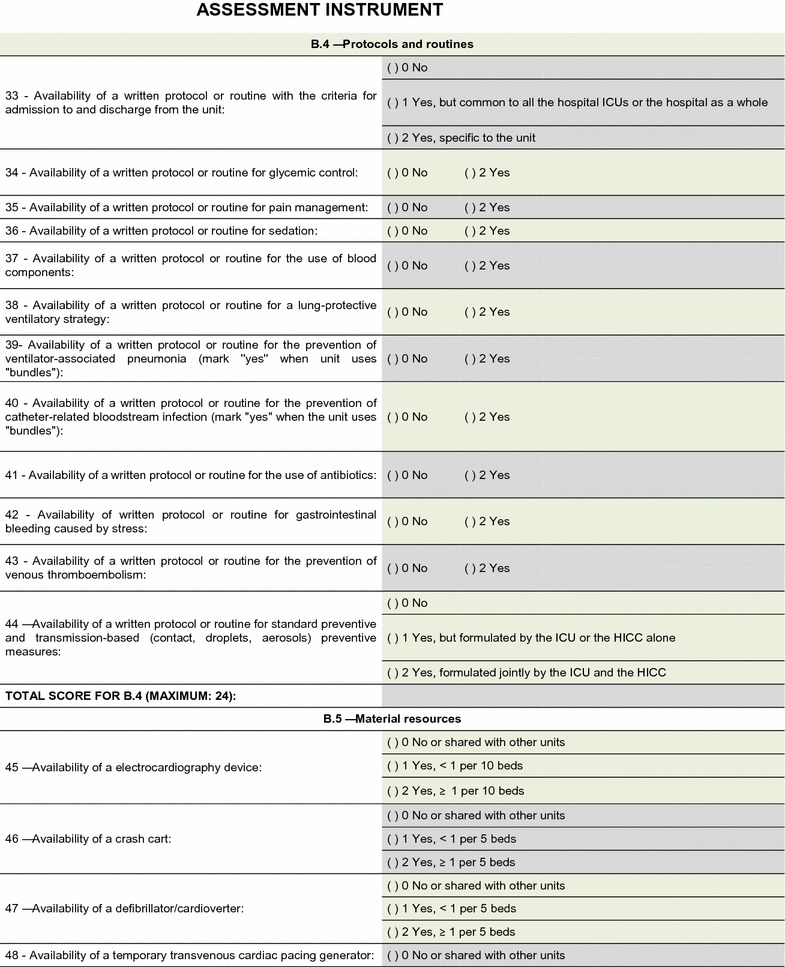
Final version of the quality assessment instrument

**Fig. 5:**
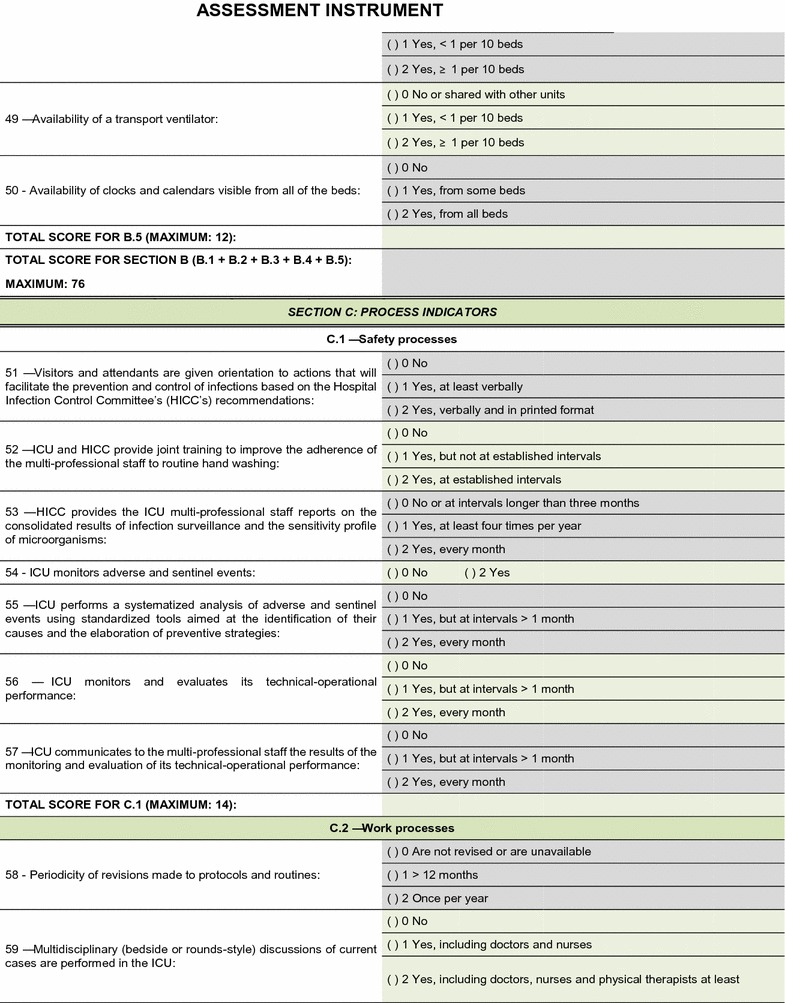
Final version of the quality assessment instrument

**Fig. 6:**
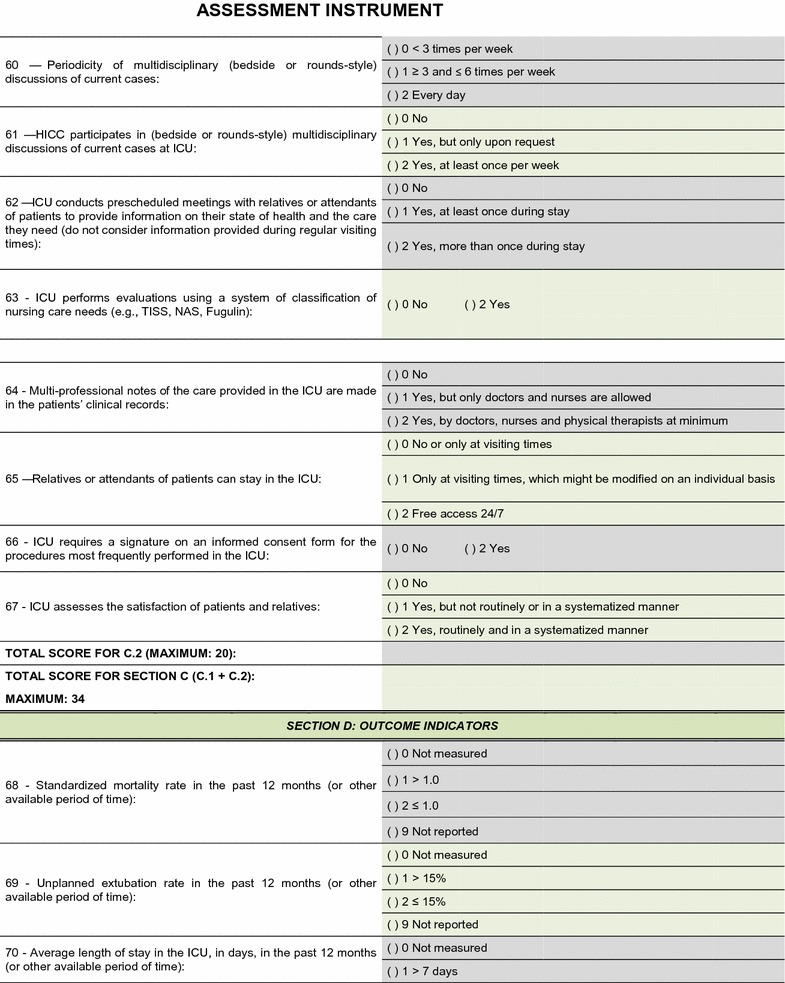
Final version of the quality assessment instrument

**Fig. 7:**
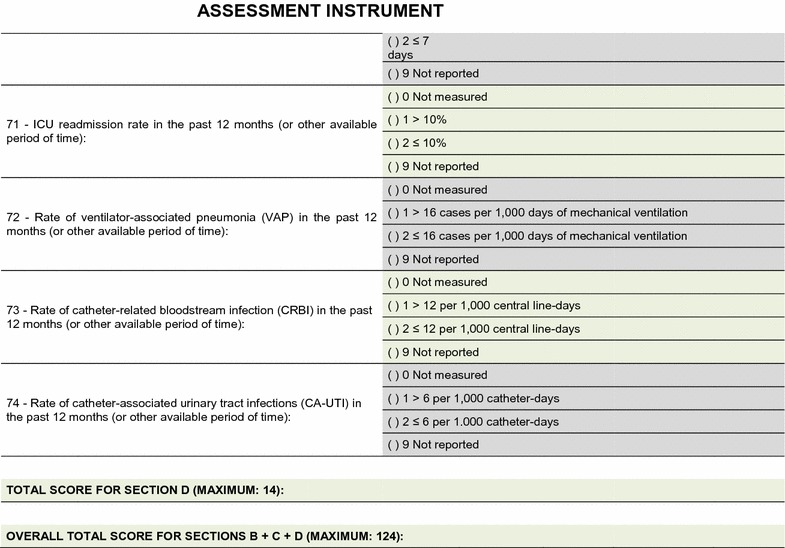
Final version of the quality assessment instrument

The field test showed that the quality assessment instrument was well accepted and easy to understand; the time needed to respond to it was 20–30 min on average. No additional inclusions or exclusions were made. The descriptions of 16 (25.8%) indicators were improved to make them more easily understandable by the interviewees and to increase the precision of the responses. The final version of the assessment tool is shown on Figs. 2, 3, 4, 5, 6, 7. The final description of the indicators is available for consultation as Additional file 1 (data not shown).

## Discussion

Compared to European countries and the US, the number of standardized instruments to evaluate healthcare services in Brazil and other developing countries is still small. However, to improve the quality of health care, tools able to measure its various dimensions accurately are necessary [21]. The present article describes the construction of an instrument for the assessment of intensive care services adapted to Brazilian norms and inclusive of indicators sanctioned in the specialized literature. In addition, new indicators based on enacted Brazilian legislation were suggested.

Quality indicators comprise one of the pillars used to make improvements in healthcare services. The systematic use of such indicators allows the detection of opportunities for improvement and deviations from pre-established standards [7]. A lack of instruments for the assessment of quality and a lack of governmental support to formulate such instruments hinder aspirations to improve the quality of health care [10].

The present study has some limitations: (a) the lack of a gold standard against which to compare the proposed instrument. (b) the application of one single approach to tool construction and data collection, whereby other techniques (field observations, reviews of clinical records, interviews with healthcare providers or users) and the participation of other actors of the health system in the tool construction are not performed. Nevertheless, valid and reliable information might also be produced when one single approach is used, and at an advantageously low cost [10]. (c) The larger number of indicators corresponding to the structure dimension compared to the process dimension, which is a function of the strong presence of norms relative to the former in Brazilian legislation. This idiosyncrasy stems from a historical concern with the instrumental quality of services at the expense of the processes. In addition, there is a great difficulty to select indicators of process that truly represent work routines in the various Brazilian regions [8]. (d) The difficulty of establishing cutoff points for the indicators that represent health care-associated infections (HAI), which is due to the lack of large-scale prevalence studies for Brazilian ICUs. (e) Possible interference in evaluation results due to interviewer or information bias. Finally, (f) “quality” is a construct, i.e. an unobservable theoretical concept and, therefore, cannot be measured directly. From this perspective, an indirect way to measure it (“proxy measure”) is the use of indicators.

Those limitations notwithstanding, the final result might be appraised positively for the following reasons: (a) the use of judicious methods in the elaboration of the instrument, including the participation of specialists in intensive care medicine and sole inclusion in the instrument of the indicators that attained 100% consensus only; (b) the inclusion of indicators already sanctioned in the international literature and the construction of others that represent the predominant norms in Brazil, thus resulting in an instrument particularly adapted to reflect the local healthcare practices; (c) the attribution of scores to the criteria, thus allowing comparison of the performance of any one service over time or to other services (*benchmarking*); (d) the inclusion of the criterion “below standard” broadening the scope of possible answers beyond mere presence or absence (“yes”, “no”) of the investigated attribute; (e) the division of the instruments into sections, which allows services to identify their weak points as well as opportunities for improvement relative to the assessed dimensions; and (f) its low cost, ease and short time required for application (30 min).

Use of interviews as an assessment instrument has been validated in low and middle-income countries. The sensitivity and specificity of that technique for assessing the quality of healthcare services are high compared to methods such as reviews of clinical records and direct observation, which are limited by missing data and interexaminer variability within this scenario. As a result, interviews provide an efficient means for assessment in countries whose healthcare system is not yet fully developed [22].

In a publication from 2008, Najjar-Pellet et al. described the construction and validation of an instrument to assess French ICUs, which included indicators of structure and process and use of a method similar to the one used for the instrument described in the present article [2]. The instrument formulated by those authors allows scores to be attributed to the assessed services, as the one described here does, but it differs from the latter in that it does not include outcome indicators.

The present document differs from the ones constructed in the Netherlands, in Germany and by ESICM in the possibility of attributing scores to the assessed services, while the latter only allow establishing the presence or absence of a given attribute, without any value judgment of it or of the final result of the evaluation of the investigated ICU.

Along with the construction of the assessment instrument, the authors sought to include the largest possible number of structure and process indicators for which there is documented scientific evidence relative to their correlation with, and impact on, the results to be studied [12]. This being the case, we call attention to the inclusion of some relevant indicators. One review performed in 2002 by Pronovost et al. showed that the mortality rate and length of stay decreased in the ICUs with 24-h availability of intensivists [17]. According to some scientific evidence, a higher number of nursing associates have better provision of healthcare. That finding might be measured by some indicators, such as a lower rate of readmissions in hospitals with larger numbers of nurses compared to the ones with lower numbers of such professionals [23].

In a publication from 2003, Pronovost et al. reported the results of a study in which rigorous methods were used and which showed that failure to use standardized processes, such as appropriate sedation, ventilator-associated pneumonia (VAP) prevention, gastrointestinal bleeding prophylaxis, venous thromboembolism (VTE) prophylaxis and appropriate use of blood transfusions, was associated with poorer outcomes, as was increased ICU length of stay and mortality [18]. Those findings give further support to the need for protocols specifically formulated for such clinical situations in ICUs, as well as to the relevance of appropriate adherence to them.

The inclusion of indicators representing a unit policy relative to the satisfaction of patients and permanence of relatives is based on some studies that reported an effect of those variables on the outcomes. Systematic assessment of satisfaction and increased presence of relatives in the ICUs indicates an improvement in the quality of care [24]. Those indicators might also be used to assess the quality of care and communication for a given unit [19, 21].

With regard to the process dimension, the assessment of daily multidisciplinary rounds for case discussion was considered to be highly relevant. Kim et al. showed that performance of such rounds is associated with lower mortality rates. The results of that study, published in 2010, are highly significant because they show that such effects occur, even in units without available intensivists. As a function of the scarcity of that type of doctor, and being that implementation of that modality of process involves little or no additional cost, performance of daily multidisciplinary rounds for case discussion is a high-impact strategy that ought to be adopted in critical care services, the ones in developing countries in particular [14].

Some articles emphasize the relevance of including specific outcome indicators in assessment instruments. In a meta-analysis published in 1997, Ashton et al. reported that the ICU readmission rate is a satisfactory indicator of the quality of processes related to the care provided over the course of hospital stays. Reduction of the quality of such processes is associated with up to a 55% increase in the risk of readmission [16].

Gastmeier et al. showed that participation in HAI surveillance systems is associated with significant reduction of their occurrence [25]. Thus, we might conclude that not only the HAI rates as such but also systematic collection of the corresponding data at participating ICUs and institutions are satisfactory quality indicators. Similarly, in a publication from 2008, Uçkay et al. asserted that the performance of indicators availability of protocols and surveillance of the prevalence of VAP in ICUs are satisfactory [13].

A constant challenge these authors all met, during the construction of the assessment instrument described here, was to keep a balance between the validity and reliability of the constructed indicators and the availability of and work load demanded by the collection of the corresponding data. One relevant issue that should be borne in mind is that in addition to aspects related to structure and process, the clinical condition of the patients admitted to the ICU exerts a strong influence on outcomes [3].

Many seemingly usefulness indicators in clinical practice were nonetheless excluded from the final version of the instrument. The reason for those exclusions was the rigorous methodological decision to include only the indicators that had achieved 100% consensus among the specialists. One further concern was to construct an instrument that would be easy to apply and take eventual regional differences into consideration.

The assessment instrument described here should be understood within a dynamic context. Consequently, we emphasize that this is a work still under construction. It is our view that, from the publication of this study, the scientific community and users would suggest new contributions that might be included in the instrument. In turn, such a move could improve its application in different scenarios. This is to say, indicators eventually shown to lose clinical relevance over time, or those no longer exhibiting variability, ought to be excluded. Conversely, new scientific evidence might come to indicate the need to include other indicators. Within that context, a systematic collection of quality indicators should not be understood as a goal unto itself but as a means to detect weak points in the system and opportunities for improvement [7]. The data thus gathered allow for planning actions aimed at the correction of the weak points detected.

## Conclusions

In the present article, we described a tool specifically constructed to assess quality of ICUs. Possible future applications of that instrument include benchmarking, multicenter studies, self-assessment of participating ICUs and assessment of the changes resulting from interventions. The instrument described here was intended to be adapted to the actual conditions in Brazil and other low and middle-income countries.

## Additional file

**Additional file 1.** Description of indicators.

## Abbreviations

ESICM: European Society of Intensive Care Medicine
HAI: health care-associated infections
ICU: intensive care unit
SCCM: Society of Critical Care Medicine
US: United States
VAP: ventilator-associated pneumonia
VTE: venous thromboembolism
