# Supplementary material for: Quality in intensive care units: proposal of an assessment instrument
Source: BMC Res Notes. 2017 Jun 26;10:222. doi: 10.1186/s13104-017-2563-3 (PMC5485612; doi:10.1186/s13104-017-2563-3)
Supplement: Supplementary file 1 — Additional file 1. Description of indicators. [file 13104_2017_2563_MOESM1_ESM.docx]

**Description of indicators**

A——Indicators of structure

A.1——Physical structure

1. Name: Availability of isolation beds
   1. Dimension: safety
   2. Definition: isolated beds should include a specific environment with a basin and a watertight cupboard for clothes and clean and dirty materials before entrance in the room (an antechamber was not deemed mandatory).
2. Equation: not applicable
3. Type: structure
4. Answer options:
   1. Standard: yes, ratio ≤ 1:10 beds
   2. Below standard: yes, ratio > 1:10 beds
   3. Out of standard: no
5. References:
   1. Resolution - RDC no. 50, February 21 2002 (2002).
6. Name: Availability of specific room for interviews with relatives or other attendants
   1. Dimension: receptivity
   2. Equation: not applicable
   3. Type: structure
   4. Answer options:
      1. Standard: yes, exclusive for this purpose
      2. Below standard: yes, multifunctional
      3. Out of standard: no
   5. References:
      1. Resolution - RDC no. 50, February 21 2002 (2002).
7. Name: Availability of the waiting room for attendants and visitors
   1. Dimension: receptivity
   2. Equation: not applicable
8. Type: structure
9. Answer options:
   - 1. Standard: yes, exclusive for the ICU
     2. Below standard: yes, shared with other services
     3. Out of standard: no
   1. References:
      1. Resolution - RDC no. 50, February 21 2002 (2002).
10. Name: Beds allow for patient privacy when needed (e.g., during baths). Observation: exclude screens.
    1. Dimension: comfort
    2. Equation: not applicable
11. Type: structure
12. Answer options:
    - 1. Standard: yes, in all beds
      2. Below standard: yes, in some beds
      3. Out of standard: no
    1. References:
       1. Resolution - RDC no. 50, February 21 2002 (2002).

A.2——Human resources

1. Name: Availability of the clinical engineering service at the hospital
   1. Dimension: safety and effectiveness
   2. Equation: not applicable
   3. Type: structure
   4. Answer options:
      1. Standard: yes, in-house
      2. Below standard: yes, outsourced
      3. Out of standard: no
   5. References:
      1. Resolution - RDC no. 50, February 21 2002 (2002).
2. Name: Availability of the medical technical manager in the ICU
   1. Dimension: effectiveness and safety
   2. Equation: not applicable
   3. Type: structure
   4. Answer options:
      1. Standard: yes, exclusive to the ICU
      2. Below standard: yes, shared with other hospital units
      3. Out of standard: no
   5. References:
      1. Resolution RDC no. 7, February 24, 2010 (2010).
3. Name: The medical technical manager is an accredited specialist in adult-intensive care medicine
   1. Dimensions: effectiveness and safety
   2. Equation: not applicable
   3. Type: structure
   4. Answer options:
      1. Standard: yes
      2. Below standard: not applicable
      3. Out of standard: no
   5. References:
      1. Resolution RDC no. 7, February 24, 2010 (2010).
      2. de Vos M, Graafmans W, Keesman E, Westert G, van der Voort PH. Quality measurement at intensive care units: which indicators should we use? J Crit Care. 2007 Dec;22(4):267-74.
4. Name: Availability of the nursing coordinator in the ICU
   1. Dimension: effectiveness and safety
   2. Equation: not applicable
   3. Type: structure
   4. Answer options:
      1. Standard: yes, exclusive to the ICU
      2. Below standard: yes, shared with other hospital units
      3. Out of standard: no
   5. References:
      1. Resolution RDC no. 7, February 24, 2010 (2010).
5. Name: Nursing coordinator participated in a specialization course or is accredited in intensive care nursing
   1. Dimension: effectiveness and safety
   2. Equation: not applicable
   3. Type: structure
   4. Answer options:
      1. Standard: yes, already completed
      2. Below standard: no, but currently attending
      3. Out of standard: no
   5. References:
      1. Resolution RDC no. 7, February 24, 2010 (2010).
6. Name: Availability of the physical therapy coordinator in the ICU
   1. Dimension: effectiveness and safety
   2. Equation: not applicable
   3. Type: structure
   4. Answer options:
      1. Standard: yes, exclusive to the ICU
      2. Below standard: yes, shared with other hospital units
      3. Out of standard: no
   5. References:
      1. Resolution RDC no. 7, February 24, 2010 (2010).
7. Name: Physical therapy coordinator participated in a specialization course or is accredited in intensive physical therapy
   1. Dimension: effectiveness and safety
   2. Equation: not applicable
   3. Type: structure
   4. Answer options:
      1. Standard: yes, already completed
      2. Below standard: no, but currently attending
      3. Out of standard: no
   5. References:
      1. Resolution RDC no. 7, February 24, 2010 (2010).
8. Name: Daily availability of regular attending physicians in the ICU
   1. Dimension: effectiveness and safety
   2. Equation: not applicable
   3. Type: structure
   4. Answer options:
      1. Standard: yes, in the day and night shifts
      2. Below standard: yes, in at least one shift
      3. Out of standard: no
   5. References:
      1. Resolution RDC no. 7, February 24, 2010 (2010).
9. Name: Regular attending physicians are accredited specialists in intensive care medicine
   1. Dimension: effectiveness and safety
   2. Equation: not applicable
   3. Type: structure
   4. Answer options:
      1. Standard: yes, all of them
      2. Below standard: yes, some
      3. Out of standard: no
   5. References:
      1. Resolution RDC no. 7, February 24, 2010 (2010).
10. Name: Ratio of regular attending physicians per bed per shift
    1. Dimension: effectiveness and safety
    2. Equation: number of regular attending physicians per shift / number of beds
    3. Type: structure
    4. Answer options:
       1. Standard: ≤ 1:10 beds
       2. Below standard: > 1:10 beds
       3. Out of standard: none
    5. References:
       1. Resolution RDC no. 7, February 24, 2010 (2010).
11. Name: Ratio of physicians on duty per bed per shift
    1. Dimension: effectiveness and safety
    2. Equation: number of physicians on duty per shift / number of beds
    3. Answer options:
       1. Standard: ≤ 1:10 beds
       2. Below standard: not applicable
       3. Out of standard: > 1:10 beds
    4. References:
       1. Resolution RDC no. 7, February 24, 2010 (2010).
12. Name: Ratio of nurses on duty per bed per shift
    1. Dimension: effectiveness and safety
    2. Equation: number of nurses on duty per shift / number of beds
    3. Type: structure
    4. Answer options:
       1. Standard: ≤ 1:10 beds
       2. Below standard: > 1:10 beds
       3. Out of standard: none
    5. References:
       1. Resolution RDC no. 7, February 24, 2010 (2010).
13. Name: Ratio of physical therapists per bed per shift
    1. Dimension: effectiveness and safety
    2. Equation: number of physical therapists per shift / number of beds
    3. Type: structure
    4. Answer options:
       1. Standard: ≤ 1:10 beds
       2. Below standard: > 1:10 beds
       3. Out of standard: none
    5. References:
       1. Resolution RDC no. 7, February 24, 2010 (2010).
14. Name: Ratio of nursing technicians per bed per shift
    1. Dimension: effectiveness and safety
    2. Equation: number of nursing technicians per shift / number of beds
    3. Type: structure
    4. Answer options:
       1. Standard: ≤ 1:2 beds
       2. Below standard: not applicable
       3. Out of standard: > 1:2 beds
    5. References:
       1. Resolution RDC no. 7, February 24, 2010 (2010).

A.3——Continued education and training

1. Name: Availability of a systematized and regular ICU-centered training program for professionals at the institution before their assignment to the unit
   1. Dimension: effectiveness and safety
   2. Equation: not applicable
   3. Type: structure
   4. Answer options:
      1. Standard: yes, for all professionals
      2. Below standard: yes, but only for some professional categories
      3. Out of standard: no
   5. References:
      1. Resolution RDC no. 7, February 24 2010 (2010).
2. Name: Availability of and regular participation in a continued education program for the multi-professional staff after being assigned to the unit
   1. Dimension: effectiveness
   2. Equation: not applicable
   3. Type: structure
   4. Answer options:
      1. Standard: yes, every month
      2. Below standard: yes, at least four times per year
      3. Out of standard: no or < 4 four times per year
   5. References:
      1. Resolution RDC no. 7, February 24, 2010 (2010).

A.4——Protocols and routines

1. Name: Availability of a written protocol or routine with the criteria for admission to and discharge from the unit
   1. Dimension: effectiveness and safety
   2. Equation: not applicable
   3. Type: structure
   4. Answer options:
      1. Standard: yes, specific to the unit
      2. Below standard: yes, common to all of the hospital units or the hospital as a whole
      3. Out of standard: no
   5. References:
      1. Rhodes A, Moreno RP, Azoulay E, Capuzzo M, Chiche JD, Eddleston J, et al. Prospectively defined indicators to improve the safety and quality of care for critically ill patients: a report from the Task Force on Safety and Quality of the European Society of Intensive Care Medicine (ESICM). Intensive Care Med. 2012 Apr;38(4):598-605.
      2. Resolution RDC no. 7, February 24, 2010 (2010).
2. Name: Availability of a written protocol or routine for glycemic control
   1. Dimension: effectiveness and safety
   2. Equation: not applicable
   3. Type: structure
   4. Answer options:
      1. Standard: yes
      2. Below standard: not applicable
      3. Out of standard: no
   5. References:
      1. de Vos M, Graafmans W, Keesman E, Westert G, van der Voort PH. Quality measurement at intensive care units: which indicators should we use? J Crit Care. 2007 Dec;22(4):267-74.
3. Name: Availability of a written protocol or routine for pain management
   1. Dimension: effectiveness
   2. Equation: not applicable
   3. Type: structure
   4. Answer options:
      1. Standard: yes
      2. Below standard: not applicable
      3. Out of standard: no
   5. References:
      1. Berenholtz SM, Dorman T, Ngo K, Pronovost PJ. Qualitative review of intensive care unit quality indicators. J Crit Care. 2002 Mar;17(1):1-12.
4. Name: Availability of a written protocol or routine for sedation
   1. Dimension: effectiveness
   2. Equation: not applicable
   3. Type: structure
   4. Answer options:
      1. Standard: yes
      2. Below standard: not applicable
      3. Out of standard: no
   5. References:
      1. Berenholtz SM, Dorman T, Ngo K, Pronovost PJ. Qualitative review of intensive care unit quality indicators. J Crit Care. 2002 Mar;17(1):1-12.
5. Name: Availability of a written protocol or routine for the use of blood components
   1. Dimension: effectiveness and safety
   2. Equation: not applicable
   3. Type: structure
   4. Answer options:
      1. Standard: yes
      2. Below standard: not applicable
      3. Out of standard: no
   5. References:
      1. Berenholtz SM, Dorman T, Ngo K, Pronovost PJ. Qualitative review of intensive care unit quality indicators. J Crit Care. 2002 Mar;17(1):1-12.
6. Name: Availability of a written protocol or routine for a lung-protective ventilatory strategy
   1. Dimension: effectiveness and safety
   2. Equation: not applicable
   3. Type: structure
   4. Answer options:
      1. Standard: yes
      2. Below standard: not applicable
      3. Out of standard: no
   5. References:
      1. Braun JP, Mende H, Bause H, Bloos F, Geldner G, Kastrup M, et al. Quality indicators in intensive care medicine: why? Use or burden for the intensivist. Ger Med Sci. 2010;8:Doc22.
7. Name: Availability of a written protocol or routine for the prevention of ventilator-associated pneumonia
   1. Dimension: effectiveness and safety
   2. Equation: not applicable
   3. Type: structure
   4. Answer options:
      1. Standard: yes
      2. Below standard: not applicable
      3. Out of standard: no
   5. References:
      1. Berenholtz SM, Dorman T, Ngo K, Pronovost PJ. Qualitative review of intensive care unit quality indicators. J Crit Care. 2002 Mar;17(1):1-12.
8. Name: Availability of a written protocol or routine for the prevention of catheter-related bloodstream infection
   1. Dimension: effectiveness and safety
   2. Equation: not applicable
   3. Type: structure
   4. Answer options:
9. Standard: yes
10. Below standard: not applicable
11. Out of standard: no
    1. References:
       1. Najjar-Pellet J, Jonquet O, Jambou P, Fabry J. Quality assessment in intensive care units: proposal for a scoring system in terms of structure and process. Intensive Care Med. 2008 Feb;34(2):278-85.
12. Name: Availability of a written protocol or routine for the use of antibiotics
    1. Dimension: effectiveness and safety
    2. Equation: not applicable
    3. Type: structure
    4. Answer options:
       1. Standard: yes
       2. Below standard: not applicable
       3. Out of standard: no
    5. References:
       1. Braun JP, Mende H, Bause H, Bloos F, Geldner G, Kastrup M, et al. Quality indicators in intensive care medicine: why? Use or burden for the intensivist. Ger Med Sci. 2010;8:Doc22.
13. Name: Availability of a written protocol or routine for gastrointestinal bleeding caused by stress
    1. Dimension: effectiveness and safety
    2. Equation: not applicable
    3. Type: structure
    4. Answer options:
       1. Standard: yes
       2. Below standard: not applicable
       3. Out of standard: no
    5. References:
       1. Berenholtz SM, Dorman T, Ngo K, Pronovost PJ. Qualitative review of intensive care unit quality indicators. J Crit Care. 2002 Mar;17(1):1-12.
14. Name: Availability of a written protocol or routine for the prevention of venous thromboembolism
    1. Dimension: effectiveness and safety
    2. Equation: not applicable
    3. Type: structure
    4. Answer options:
       1. Standard: yes
       2. Below standard: not applicable
       3. Out of standard: no
    5. References:
       1. Berenholtz SM, Dorman T, Ngo K, Pronovost PJ. Qualitative review of intensive care unit quality indicators. J Crit Care. 2002 Mar;17(1):1-12.
15. Name: Availability of a written protocol or routine for standard preventive and transmission-based (contact, droplets, aerosols) preventive measures formulated in conjunction with the Hospital Infection Control Committee (HICC)
    1. Dimension: safety
    2. Equation: not applicable
    3. Type: structure
    4. Answer options:
       1. Standard: yes, formulated together with the HICC
       2. Below standard: yes, formulated by the unit or the HICC alone
       3. Out of standard: no
    5. References:
       1. Resolution RDC no. 7, February 24, 2010 (2010).

A.5——Material resources

1. Name: Availability of an electrocardiography device
   1. Dimension: effectiveness
   2. Equation: not applicable
   3. Type: structure
   4. Answer options:
      1. Standard: yes, ≤ 1 per 10 beds
      2. Below standard: yes, > 1 per 10 beds
      3. Out of standard: no or shared with other units
   5. References:
      1. Resolution RDC no. 7, February 24, 2010 (2010).
2. Name: Availability of a crash cart
   1. Dimension: effectiveness and safety
   2. Equation: not applicable
   3. Type: structure
   4. Answer options:
      1. Standard: yes, ≤ 1 per 5 beds
      2. Below standard: yes, > 1 per 5 beds
      3. Out of standard: no or shared with other units
   5. References:
      1. Resolution RDC no. 7, February 24, 2010 (2010).
3. Name: Availability of a defibrillator/cardioverter
   1. Dimension: effectiveness and safety
   2. Equation: not applicable
   3. Type: structure
   4. Answer options:
      1. Standard: yes, ≤ 1 per 5 beds
      2. Below standard: yes, > 1 per 5 beds
      3. Out of standard: no or shared with other units
   5. References:
      1. Resolution RDC no. 7, February 24, 2010 (2010).
4. Name: Availability of a temporary transvenous cardiac pacing generator
   1. Dimension: effectiveness and safety
   2. Equation: not applicable
   3. Type: structure
   4. Answer options:
      1. Standard: yes, ≤ 1 per 10 beds
      2. Below standard: yes, > 1 per 10 beds
      3. Out of standard: no or shared with other units
   5. References:
      1. Resolution RDC no. 7, February 24, 2010 (2010).
5. Name: Availability of a transport ventilator
   1. Dimension: effectiveness and safety
   2. Equation: not applicable
   3. Type: structure
   4. Answer options:
      1. Standard: yes, ≤ 1 per 10 beds
      2. Below standard: yes, > 1 per 10 beds
      3. Out of standard: no or shared with other units
   5. References:
      1. Resolution RDC no. 7, February 24, 2010 (2010).
6. Name: Availability of clocks and calendars visible from all of the beds
   1. Dimension: comfort and humanization
   2. Equation: not applicable
   3. Type: structure
   4. Answer options:
      1. Standard: yes, from all of the beds
      2. Below standard: yes, from some of the beds
      3. Out of standard: no
   5. References:
      1. Resolution RDC no. 7, February 24, 2010 (2010).

B—Process indicators

B.1—Safety processes

1. Name: Visitors and attendants are given orientation to actions that facilitate the prevention and control of infections based on the HICC’s recommendations
   1. Dimension: safety
   2. Equation: not applicable
   3. Type: process
   4. Answer options:
      1. Standard: yes, verbally and in printed format
      2. Below standard: yes, at least verbally
      3. Out of standard: no
   5. References:
      1. Resolution RDC no. 7, February 24, 2010 (2010).
2. Name: ICU and HICC provide joint training to improve the multi-professional staff’s adherence to routine hand washing
   1. Dimension: safety
   2. Equation: not applicable
   3. Type: process
   4. Answer options:
      1. Standard: yes, at established intervals
      2. Below standard: yes, but not at established intervals
      3. Out of standard: no
   5. References:
      1. Resolution RDC no. 7, February 24, 2010 (2010).
3. Name: HICC communicates to the ICU multi-professional staff the results of infection surveillance and the sensitivity profile of microorganisms
   1. Dimension: safety
   2. Equation: not applicable
   3. Type: process
   4. Answer options:
      1. Standard: yes, every month
      2. Below standard: yes, at least four times per year
      3. Out of standard: no, or at intervals longer than three months
   5. References:
      1. Resolution RDC no. 7, February 24, 2010 (2010).
4. Name: ICU monitors adverse and sentinel events
   1. Dimension: safety
   2. Equation: not applicable
   3. Type: process
   4. Answer options:
      1. Standard: yes
      2. Below standard: not applicable
      3. Out of standard: no
   5. References:
      1. Rhodes A, Moreno RP, Azoulay E, Capuzzo M, Chiche JD, Eddleston J, et al. Prospectively defined indicators to improve the safety and quality of care for critically ill patients: a report from the Task Force on Safety and Quality of the European Society of Intensive Care Medicine (ESICM). Intensive Care Med. 2012 Apr;38(4):598-605.
      2. Najjar-Pellet J, Jonquet O, Jambou P, Fabry J. Quality assessment in intensive care units: proposal for a scoring system in terms of structure and process. Intensive Care Med. 2008 Feb;34(2):278-85.
      3. Resolution RDC no. 7, February 24, 2010 (2010).
5. Name: ICU performs a systematic analysis of adverse and sentinel events aimed at the identification of their causes and the elaboration of preventive strategies
   1. Dimension: safety
   2. Equation: not applicable
   3. Type: process
   4. Answer options:
      1. Standard: yes, every month
      2. Below standard: yes, but not at established intervals
      3. Out of standard: no
   5. References:
      1. Resolution RDC no. 7, February 24, 2010 (2010).
6. Name: ICU performs evaluations of its technical performance
   1. Dimension: safety and effectiveness
   2. Equation: not applicable
   3. Type: process
   4. Answer options:
      1. Standard: yes, every month
      2. Below standard: yes, but at intervals > 1 month
      3. Out of standard: no
   5. References:
      1. Resolution RDC no. 7, February 24, 2010 (2010).
      2. Normative Instruction no. 4. Sect. 1 (2010).
7. Name: ICU communicates to the multi-professional staff the results of the evaluations of its technical performance
   1. Dimension: safety and effectiveness
   2. Equation: not applicable
   3. Type: process
   4. Answer options:
      1. Standard: yes, every month
      2. Below standard: yes, but at intervals > 1 month
      3. Out of standard: no
   5. References:
      1. Resolution RDC no. 7, February 24, 2010 (2010).
      2. Normative Instruction no. 4. Sect. 1 (2010).

B.2—Work processes

1. Name: Periodicity of the revision of protocols and routines
   1. Dimension: effectiveness and safety
   2. Equation: not applicable
   3. Type: process
   4. Answer options:
      1. Standard: once per year
      2. Below standard: > 12 months
      3. Out of standard: No revision is performed, or protocols are not available
   5. References:
      1. Resolution RDC no. 7, February 24, 2010 (2010).
2. Name: Multidisciplinary discussions of current cases are performed in the ICU
   1. Dimension: effectiveness and safety
   2. Equation: not applicable
   3. Type: process
   4. Answer options:
      1. Standard: yes, including doctors, nurses, and physical therapists at minimum
      2. Below standard: yes, including doctors and nurses
      3. Out of standard: no
   5. References:
      1. Rhodes A, Moreno RP, Azoulay E, Capuzzo M, Chiche JD, Eddleston J, et al. Prospectively defined indicators to improve the safety and quality of care for critically ill patients: a report from the Task Force on Safety and Quality of the European Society of Intensive Care Medicine (ESICM). Intensive Care Med. 2012 Apr;38(4):598-605.
3. Name: Periodicity of multidisciplinary discussions of current cases
   1. Dimension: effectiveness and safety
   2. Equation: not applicable
   3. Type: process
   4. Answer options:
      1. Standard: every day
      2. Below standard: ≥ 3 and ≤ 6 times per week
      3. Out of standard: < 3 times per week
   5. References:
      1. Rhodes A, Moreno RP, Azoulay E, Capuzzo M, Chiche JD, Eddleston J, et al. Prospectively defined indicators to improve the safety and quality of care for critically ill patients: a report from the Task Force on Safety and Quality of the European Society of Intensive Care Medicine (ESICM). Intensive Care Med. 2012 Apr;38(4):598-605.
4. Name: HICC participates in rounds and discussions of current cases in the ICU
   1. Dimension: safety
   2. Equation: not applicable
   3. Type: process
   4. Answer options:
      1. Standard: yes, at least once per week
      2. Below standard: yes, but only upon request
      3. Out of standard: no
   5. References:
      1. Resolution RDC no. 7, February 24, 2010 (2010).
5. Name: ICU conducts prescheduled meetings with relatives or attendants of patients to provide information on their state of health and the care they need
   1. Dimension: satisfaction
   2. Definition: prescheduled multidisciplinary meetings with the patients’ relatives in addition to the information provided daily about the patients’ state of health or clinical progression. Staff/family interaction meetings.
   3. Equation: not applicable
   4. Type: process
   5. Answer options:
      1. Standard: yes, more than once during the patient’s stay in the ICU
      2. Below standard: yes, at least once during the patient’s stay in the ICU
      3. Out of standard: no
   6. References:
      1. Martín MC, Gil CL, de la Hoz JC, Herrejón EP, Sáez FN, Varela JB, et al. Sociedad Española de Medicina Intensiva Crítica y Unidades Coronarias - Indicadores de Calidad en el Enfermo Crítico Actualización 2011 [Spanish society of critical intensive medicine and coronary care units - quality indicators in critical patients. Update 2011]. 2011.
      2. Resolution RDC no. 7, February 24, 2010 (2010).
6. Name: ICU performs evaluations using the System of Classification of Nursing Care Needs
   1. Dimension: effectiveness and safety
   2. Equation: not applicable
   3. Type: process
   4. Answer options:
      1. Standard: yes
      2. Below standard: not applicable
      3. Out of standard: no
   5. References:
      1. Resolution RDC no. 7, February 24, 2010 (2010).
7. Name: Multi-professional notes of the care provided at the ICU are made in the patients’ clinical records
   1. Dimension: effectiveness and safety
   2. Equation: not applicable
   3. Type: process
   4. Answer options:
      1. Standard: yes, by doctors, nurses, and physical therapists at minimum
      2. Below standard: yes, but only doctors and nurses are allowed to do so
      3. Out of standard: no
   5. References:
      1. Resolution RDC no. 7, February 24, 2010 (2010).
8. Name: Policy for patients’ relatives or attendants staying in the ICU
   1. Dimension: satisfaction
   2. Equation: not applicable
   3. Type: process
   4. Answer options:
      1. Standard: free access 24/7
      2. Below standard: only at visiting times, which might be modified on an individual basis
      3. Out of standard: at visiting times only
   5. References:
      1. Resolution RDC no. 7, February 24, 2010 (2010).
      2. Souza SROS, Silva CA, Mello ÚM, Ferreira CN. Aplicabilidade de indicador de qualidade subjetivo em Terapia Intensiva. Revista Brasileira de Enfermagem [Applicability of a subjective quality indicator in intensive care]. 2006;59:201-5.
      3. Neves FBCS, Dantas MP, Bitencourt AGV, Vieira PS, Magalhães LT, Teles JMM, et al. Análise da satisfação dos familiares em unidade de terapia intensiva [Analysis of family satisfaction in the intensive care unit]. Revista Brasileira de Terapia Intensiva. 2009;21:32-7.
9. Name: ICU requires a signature on an informed consent form for the procedures most frequently performed in the ICU
   1. Dimension: safety and effectiveness
   2. Equation: not applicable
   3. Type: process
   4. Answer options:
      1. Standard: yes
      2. Below standard: not applicable
      3. Out of standard: no
   5. References:
      1. Resolution RDC no. 7, February 24, 2010 (2010).
10. Name: ICU assesses the satisfaction of patients and relatives
    1. Dimension: satisfaction
    2. Equation: not applicable
    3. Type: process
    4. Answer options:
       1. Standard: yes, routinely and in a systematized manner
       2. Below standard: yes, but not routinely or in a systematized manner
       3. Out of standard: no
    5. References:
       1. Berenholtz SM, Dorman T, Ngo K, Pronovost PJ. Qualitative review of intensive care unit quality indicators. J Crit Care. 2002 Mar;17(1):1-12.
       2. de Vos M, Graafmans W, Keesman E, Westert G, van der Voort PH. Quality measurement at intensive care units: which indicators should we use? J Crit Care. 2007 Dec;22(4):267-74.
       3. Souza SROS, Silva CA, Mello ÚM, Ferreira CN. Aplicabilidade de indicador de qualidade subjetivo em Terapia Intensiva [Applicability of a subjective quality indicator in intensive care]. Revista Brasileira de Enfermagem. 2006;59:201-5.
       4. Neves FBCS, Dantas MP, Bitencourt AGV, Vieira PS, Magalhães LT, Teles JMM, et al. Análise da satisfação dos familiares em unidade de terapia intensiva [Analysis of family satisfaction in the intensive care unit]. Revista Brasileira de Terapia Intensiva. 2009;21:32-7.

C—Outcome indicators

1. Name: Standardized mortality ratio in the past 12 months (or other available period of time)
   1. Dimension: safety
   2. Equation: number of deaths in the assessed period of time / number of expected deaths in the assessed period of time
   3. Type: outcome
   4. Answer options
      1. Standard: ≤ 1.0
      2. Below standard: > 1.0
      3. Out of standard: not measured
      4. Not reported
   5. References:
      1. de Vos M, Graafmans W, Keesman E, Westert G, van der Voort PH. Quality measurement at intensive care units: which indicators should we use? J Crit Care. 2007 Dec;22(4):267-74.
      2. Rhodes A, Moreno RP, Azoulay E, Capuzzo M, Chiche JD, Eddleston J, et al. Prospectively defined indicators to improve the safety and quality of care for critically ill patients: a report from the Task Force on Safety and Quality of the European Society of Intensive Care Medicine (ESICM). Intensive Care Med. 2012 Apr;38(4):598-605.
2. Name: Unplanned extubation rate in the past 12 months (or other available period of time)
   1. Dimension: safety
   2. Equation: (number of unplanned extubations in the assessed period of time / number of intubated patients in the assessed period of time) x 100
   3. Type: outcome
   4. Answer options:
      1. Standard: ≤ 15%
      2. Below standard: > 15%
      3. Out of standard: not measured
      4. Not reported
   5. References:
      1. de Vos M, Graafmans W, Keesman E, Westert G, van der Voort PH. Quality measurement at intensive care units: which indicators should we use? J Crit Care. 2007 Dec;22(4):267-74.
      2. Rhodes A, Moreno RP, Azoulay E, Capuzzo M, Chiche JD, Eddleston J, et al. Prospectively defined indicators to improve the safety and quality of care for critically ill patients: a report from the Task Force on Safety and Quality of the European Society of Intensive Care Medicine (ESICM). Intensive Care Med. 2012 Apr;38(4):598-605.
3. Name: Name: Average length of stay in the ICU, in days, in the past 12 months (or other available period of time)
   1. Dimension: safety
   2. Definition: average length of stay in the ICU, in days
   3. Equation: number of patients in a given period of time / number of patients discharged from the unit during the same period of time
   4. Type: outcome
   5. Answer options:
      1. Standard: ≤ 7 days
      2. Below standard: > 7 days
      3. Out of standard: not measured
      4. Not reported
   6. References:
      1. Berenholtz SM, Dorman T, Ngo K, Pronovost PJ. Qualitative review of intensive care unit quality indicators. J Crit Care. 2002 Mar;17(1):1-12.
      2. de Vos M, Graafmans W, Keesman E, Westert G, van der Voort PH. Quality measurement at intensive care units: which indicators should we use? J Crit Care. 2007 Dec;22(4):267-74.
      3. Resolution RDC no. 7, February 24, 2010 (2010).
4. Name: ICU readmission rate in the past 12 months (or other available period of time)
   1. Dimension: safety
   2. Definition: unplanned ICU readmission rate corresponding to patients discharged from the ICU but having stayed in the hospital
   3. Equation: (number of readmissions in the assessed period of time / total number of admissions in the same period of time ) x 100
   4. Type: outcome
   5. Answer options:
      1. Standard: ≤ 10%
      2. Below standard: > 10%
      3. Out of standard: not measured
      4. Not reported
   6. References:
      1. Berenholtz SM, Dorman T, Ngo K, Pronovost PJ. Qualitative review of intensive care unit quality indicators. J Crit Care. 2002 Mar;17(1):1-12.
      2. Rhodes A, Moreno RP, Azoulay E, Capuzzo M, Chiche JD, Eddleston J, et al. Prospectively defined indicators to improve the safety and quality of care for critically ill patients: a report from the Task Force on Safety and Quality of the European Society of Intensive Care Medicine (ESICM). Intensive Care Med. 2012 Apr;38(4):598-605.
      3. Resolution RDC no. 7, February 24, 2010 (2010).
5. Name: Rate of ventilator-associated pneumonia (VAP) in the past 12 months (or other available period of time)
   1. Dimension: safety
   2. Equation: number of VAP episodes / total number of days under mechanical ventilation—MV) x 1,000 days of MV
   3. Type: outcome
   4. Answer options:
      1. Standard: ≤ 16 cases per 1,000 days of MV
      2. Below standard: > 16 cases per 1,000 days of MV
      3. Out of standard: not measured
      4. Not reported
   5. References:
      1. Resolution RDC no. 7, February 24, 2010 (2010).
6. Name: Rate of catheter-related bloodstream infection (CRBI) in the past 12 months (or other available period of time)
   1. Dimension: safety
   2. Equation: number of new cases of CRBI during the assessed period of time / number of patients with central line-days during the same period of time) x 1,000
   3. Type: outcome
   4. Answer options:
      1. Standard: ≤ 12 per 1,000 central line-days
      2. Below standard: > 12 per 1,000 central line-days
      3. Out of standard: not measured
      4. Not informed
   5. References:
      1. Berenholtz SM, Dorman T, Ngo K, Pronovost PJ. Qualitative review of intensive care unit quality indicators. J Crit Care. 2002 Mar;17(1):1-12.
      2. Rhodes A, Moreno RP, Azoulay E, Capuzzo M, Chiche JD, Eddleston J, et al. Prospectively defined indicators to improve the safety and quality of care for critically ill patients: a report from the Task Force on Safety and Quality of the European Society of Intensive Care Medicine (ESICM). Intensive Care Med. 2012 Apr;38(4):598-605.
      3. Martín MC, Gil CL, de la Hoz JC, Herrejón EP, Sáez FN, Varela JB, et al. Sociedad Española de Medicina Intensiva Crítica y Unidades Coronarias - Indicadores de Calidad en el Enfermo Crítico Actualización 2011 [Spanish society of critical intensive medicine and coronary care units - quality indicators in critical patients. Update 2011]. 2011.
      4. Normative Instruction no. 4. Sect. 1 (2010).
      5. Lobo RD, Levin AS, Oliveira MS, Gomes LM, Gobara S, Park M, et al. Evaluation of interventions to reduce catheter-associated bloodstream infection: continuous tailored education versus one basic lecture. Am J Infect Control. 2010 Aug;38(6):440-8.
7. Name: Rate of catheter-associated urinary tract infections (CA-UTI) in the past 12 months (or other available period of time)
   1. Dimension: safety
   2. Equation: (number of CA-UTI episodes / number of catheter-days) x 1,000
   3. Type: outcome
   4. Answer options:
      1. Standard: ≤ 6 per 1,000 catheter-days
      2. Below standard: > 6 per 1,000 catheter-days
      3. Out of standard: not measured
      4. Not reported
   5. References:
      1. Normative Instruction n. 4. Sect. 1 (2010).
